# Comparison of CAD/CAM and conventional microvascular free bone flaps in reconstructive head and neck surgery: a retrospective analysis of over 110 cases

**DOI:** 10.1007/s00784-025-06573-1

**Published:** 2025-11-03

**Authors:** Jonas Wüster, Pit Jacob Voss, Stella Magdalena Tunn, Julian Brauchle, Rainer Schmelzeisen, Marc Christian Metzger, Marc Anton Fuessinger, Leonard Simon Brandenburg

**Affiliations:** 1 Department of Oral and Maxillofacial Surgery, Faculty of Medicine, Medical Center-University of Freiburg, University of Freiburg, Hugstetterstr. 55, 79106 Freiburg, Germany; 2 Institute of STEM Education, St. Gallen University of Teacher Education, St. Gallen, Switzerland

**Keywords:** Head and neck cancer, CAD/CAM, Patient specific implant, Free flaps, Osseous reconstruction

## Abstract

**Objectives:**

Computer-Aided Design and Computer-Aided Manufacturing (CAD/CAM) – techniques are increasingly considered the gold standard for orofacial osseous reconstruction. By facilitating the harvesting and transfer of microvascular bone flaps, CAD/CAM offers potential advantages over conventional methods. Therefore, this study aimed to compare both methods in a large cohort.

**Materials and methods:**

Patients who underwent conventional or CAD/CAM-planned osseous reconstruction with microvascular free flaps between 2000 and 2019 were included. Primary outcomes included osseous union, plate fracture, duration of intensive care (ICU) treatment, ischemic time, surgical time, oncological clearance and red blood cell transfusion requirements (RBT). Confounding factors influencing primary results were also analyzed. Statistical analyses were performed with a multivariate regression model; the level of significance was set to *p* = 0.050.

**Results:**

A total of 112 patients were included, with 56 cases undergoing conventional reconstruction and 56 receiving CAD/CAM-assisted surgery. Patients in the CAD/CAM-group had significantly shorter ischemic times (*p* = 0.007), with no disadvantages regarding resection margins (*p* = 0.509). Nevertheless, reconstruction with scapula osteocutaneous free flaps (SOFF) and reconstruction with deep circumflex iliac artery grafts (DCIA) flaps showed a significant association (*p* = 0.015 and *p* = 0.036 respectively) for red blood cell transfusion requirements when performed with CAD/CAM.

**Conclusions:**

CAD/CAM-techniques reduce ischemic time in osseous reconstruction and show no disadvantages regarding clear margin resection. When performing complex reconstructions with chimeric SOFF using CAD/CAM-technique, longer ICU stay and prolonged surgical time was noticed. Furthermore, CAD/CAM-technique is associated with higher requirements for RBT when performing reconstructions with SOFF and DCIA flaps. Nevertheless, these results must be interpreted with caution, as the conventional group included younger patients (mean: 58.66 ± 9.15 years) than the CAD/CAM group (mean: 64.98 ± 10.41 years).

**Clinical relevance:**

CAD/CAM-techniques reveal advantages, such as reduced ischemic time when compared to the conventional technique and enables extensive reconstructive surgery even for older patients. Nevertheless, due to the retrospective character with heterogeneous study groups, further research is highly recommended to evaluate long-term outcomes and further define the role of CAD/CAM in oncologic head and neck reconstruction.

## Introduction

 Head and neck cancers (HNC) are the seventh most common tumors worldwide, including all epithelial malignancies of the oral cavity, pharynx (subdivision into naso-, oro-, and hypopharynx), larynx, nasal cavity, paranasal sinus, and salivary glands [1, 2]. Within this heterogeneous group, the incidence of oral tumors, especially oral squamous cell carcinoma (OSCC), is continuously increasing and is considered a global health issue [3, 4]. Especially in OSCC, the surgical therapy often requires osseous resection when the tumor is in direct vicinity of the mandible, resulting in a segmental mandibulectomy [5]. This generally causes extensive osseus defects that exceed the intrinsic bone healing capacity [6]. Therefore, primary or secondary osseous reconstruction is generally carried out in these cases [7]. Despite ongoing advances in Bone Tissue Engineering with even new insight into the microstructure of bone graft and cell morphometry [8–10], autologous bone grafts are still regarded the “gold standard” in osseous reconstruction [11, 12].

There are several options for osseous reconstruction of the head and neck, whereby the deep circumflex iliac artery (DCIA) grafts, free fibula flaps (FFF) and scapula osteocutaneous free flaps (SOFF) are most commonly used [13]. Since the introduction of microvascular surgery, surgical techniques are continuously evolving. In the last decades the rapid developments in imaging and medical computer applications brought up computer-aided design and computer-assisted manufacturing (CAD/CAM)-technology [14]. Recently, the integration of CAD/CAM guides and plates in reconstructive head and neck surgery has revolutionized osseous reconstruction [15, 16]. For the CAD/CAM-technique, high-resolution computed tomography (CT)-scans are evaluated preoperatively, and the planned procedure is conducted virtually prior to the actual surgery [17]. To transfer the virtual plan into the operation theatre, surgical guides and patient specific implants (PSI) are manufactured using 3D-printers and selective laser melting [18, 19]. The surgical navigation supplied by surgical guides alleviates surgery, as intraoperative bending of conventional osteosynthesis plates and re-shaping of the graft is not required [20]. The anatomically designed PSI enables anatomical fitting of the osteosynthesis plate and can be fixated in a fast manner using pre-drilled screwholes. This advancement has resulted in highly accurate reconstructions, reduced surgical time, and improved aesthetic and functional outcomes [21, 22]. In this context it was described that PSIs guarantee higher stability of osteosynthesis as they do not need to be bend [23, 24]. Moreover, some studies describe shorter hospitalization in CAD/CAM-cases, a decreased surgical time and reduced ischemic time of the graft [25, 26].

As CAD/CAM is used routinely for approximately a decade in most departments [27, 28], a relatively high number of studies on CAD/CAM-techniques in reconstructive head and neck surgery exist. However, most of these studies report on small cohorts, including about 10 CAD/CAM-cases on average per article [28, 29]. This study aims to provide a more comprehensive analysis by comparing patients who underwent osseous reconstruction of the head and neck region using either conventional methods or CAD/CAM-fabricated bone grafts in a large cohort of over 100 cases. Outcome, such as osseous union and plate fracture were evaluated, with a particular focus on identifying potential influencing factors.

## Methods

### Ethical statement

This study was approved by the ethics committee of the Albert-Ludwigs-University Freiburg, Germany (No. 30/20) and was registered in the German Clinical Trials register (No. DRKS00029159).

### Patient consent statement

In accordance with the statement of the ethics committee, individual consent of the patients was not necessary as the data was processed retrospectively after anonymization.

### Inclusion and exclusion criteria

For this retrospective non-interventional study, patients who underwent microsurgical reconstruction of the oral cavity due to tumor resection at the Clinic of Oral and Maxillofacial Surgery, Medical Center - University of Freiburg, Germany between 2000 and 2019 were considered for study inclusion. Patients with microvascular transfer of soft tissue only were excluded from the study, because CAD/CAM is not applicable in these cases. Only patients who received microvascular transplantation of osseus grafts were further investigated. Cases with DCIA grafts, FFF, and SOFF were included. All flaps were further characterized: if a skin paddle was used (for DCIA and FFF: yes/no) and if the SOFF was raised as a chimeric flap including the latissimus dorsi (LD). Only patients with legal age were enrolled in this study.

### Clinical evaluation

The ischemic time was measured in minutes and recorded as from ligation of the artery to the time of successful restoration of arterial blood flow (single stitches with Ethilon 8 − 0, Ethicon, Johnson & Johnson, Edinburgh, Scotland, Great Britain) and venous return via the pedicle vein. Cases in which blood flow had to be subsequently interrupted for additional suturing or revision of one of the anastomoses were excluded because the ischemic time could not be clearly determined. Osseous union was investigated using the available postoperative imaging, which was conducted at least 12 months after surgery. If 3D- and 2D-imaging was available, only the 3D dataset was evaluated. Osseous union was defined according to Rendenbach et al. whereby osseous union was classified as subtotal when the investigators rated at least one of the gaps between flap segments or between the mandible and flap to have an osseous contact area of less than 50% [30].

### Data acquisition

Medical records were reviewed retrospectively, using electronic patient charts. Out of this group, we divided all patients into subgroups according to surgical technique (CAD/CAM vs. conventional). Additionally, to osseous union/subtotal osseous union and ischemic time, we identified several patient-specific factors to test the influence of potential confounding factors (see below). The collected data was arranged using a Microsoft Excel spreadsheet (Microsoft Excel^®^ Version 16.0, Microsoft Corporation, Albuquerque, NM, USA).

### Primary results

The following parameters were primarily compared regarding statistical significant differences between the conventional and the CAD/CAM-group: operative time (in hours), ischemic time (in minutes), osseous union (yes/no), duration of intensive care treatment (in days), requirement of red blood cell transfusion (yes/no), oncological clearance (R0 vs. R1/2) and plate fracture (yes/no).

### Confounders

Confounding variables were defined as patient specific factors, which might have an impact on primary results. The following variables were investigated as potential confounding factors: adjuvant radiation (yes/no), graft type (DCIA vs. FFF vs. SOFF), smoking (yes/no) and American Society of Anesthesiology Physical Status (ASA) classification. Additionally, the UICC status was included as a control variable on the length of ICU stay and duration of surgery. Furthermore, in each model, patient age was included as a control variable.

### Statistics

Due to documentation gaps in the performed surgeries, the data set contains a total of 7.14% missing values. These missing values appear primarily in the variables ischemic time and osseous union. To avoid bias in the analyses [31] and to increase the number of retained cases, the missing values were imputed using model-based multiple imputation [32] in “Blimp” [33]. The imputation process accounted for the full structure of the computed model to improve precision by running 5000 iterations. The quality of the imputation was assessed using posterior predictive checking and the potential scale reduction factor ($\:\widehat{R\:}$< 1.01).

To examine the effects on the primary results, multivariate regression analyses were performed in “MPlus” [34] using bootstrapping. In this approach, multiple outcome variables are explained by a set of predictors or confounders. The relationships and variances are modeled simultaneously, while controlling for the correlations among the included dependent variables [31]. Bootstrapping was used to improve robustness by repeatedly resampling the dataset with replacement. This approach enhances inference reliability given the model complexity, the presence of multiple dependent variables, and the available sample size. The distinction between conventionally performed surgeries (coded as 0) and surgeries using CAD/CAM (coded as 1) was used as the independent variable in the regression model. The influence of each confounder on the primary results was assessed individually as a moderator of the effect of the independent variable on the dependent variables. Data preparation and dummy coding of categorical variables were performed in “R” [35]. The significance level was set to *p* = 0.050. For descriptive analyses, relative frequencies, means, medians, and standard deviations were computed. Prior to analysis, a G*Power analysis was performed to confirm that the sample size was sufficient to detect the expected effect sizes with a statistical power of 0.95 [32]. These a priori analyses confirmed the adequacy of the sample size for the planned regression analysis.

## Results

### Study group characteristics

Initially 401 patients receiving orofacial reconstruction with a microvascular transplant were found. Of these, 289 patients were excluded, because they received a soft tissue graft without bone. A total of 112 patients (66 men (58.9%) and 46 women (41.1%)) were included as they received a microvascular bone graft. Table 1 gives an overview of the different types of used microvascular grafts. Within this group, CAD/CAM-surgery was performed in 56 cases and conventional surgery in 56 cases. The mean follow-up time was 4.04 years ± 5.07 years (conventional: 7.03 ± 5.63 years; CAD/CAM: 1.04 ± 1.32 years), and the mean age was 61.82 years ± 10.29 years (conventional: 58.66 ± 9.15 years; CAD/CAM: 64.98 ± 10.41 years) (Table 2). In 106 patients, osseous reconstruction was required due to resection of OSCC (OSCC: 106/112) and in 6 due to resection of a sarcoma (Sarcoma: 6/112). For all 112 patients ASA classification was collected, whereby 1 patient was classified as ASA I (ASA I: 1/112), 35 patients as ASA II (ASA II: 35/112), 73 patients as ASA III (ASA III: 73/112), and 3 patients as ASA IV (ASA IV: 3/112) (Table 2). Patients with ASA III/IV classification showed a longer ICU stay than patients with ASA I/II classification (5.75 ± 3.97 days vs. 4.03 ± 3.99 days). A total of 92 surgeries (*n* = 92/112) were performed in the time between 2012 and 2019, with a significantly higher (*p* < 0.001) follow-up time in the conventional group (7.03 ± 5.63 years) than in the CAD/CAM group (1.04 ± 1.32 years). At the same time, patients receiving CAD/CAM-surgery were significantly older (*p* = 0.002) (64.98 ± 10.41 years) compared to patients who received conventional surgery (58.66 ± 9.15 years).

**Table 1:** Distribution of all flap types

Grafts	**Total number**	**CAD/CAM Surgery **	**Conventional Surgery**	**Subtotal osseous union**	**Plate fracture **
All	112	56 *(n = 56/112)*	56 *(n = 56/112)*	14 *(n = 14/78)*	9 *(n = 9/112)*
DCIA	43 *(n = 43/112)*	31 *(n = 31/56)*	12 *(n = 12/56)*	5 *(n = 5/14)*	6 *(n = 6/9)*
*DCIA with skin paddle*	29 *(n = 29/43)*	19 *(n = 19/31)*	10 *(n = 10/12)*	*5* *(n = 5/5)*	3 *(n = 3/6)*
*DCIA without skin paddle*	14 *(n = 14/43)*	12 *(n = 12/31)*	2 *(n = 2/12)*	*0* *(n = 0/5)*	*3* *(n = 3/6)*
FFF	40 *(n = 40/112)*	13 *(n = 13/56)*	27 *(n = 27/56)*	3 *(n = 3/14)*	3 *(n = 3/9)*
*FFF with skin paddle*	16 *(n = 16/40)*	6 *(n = 6/13)*	10 *(n = 10/27)*	1 *(n = 1/3)*	0 *(n = 0/3)*
*FFF without skin paddle*	24 *(n = 24/40)*	7 *(n = 7/13)*	17 *(n = 17/27)*	2 *(n = 2/3)*	3 *(n = 3/3)*
SOFF	29 *(n = 29/112)*	12 *(n = 12/56)*	17 *(n = 17/56)*	6 *(n = 6/14)*	0 *(n = 0/9)*
*Non-chimeric SOFF*	6 *(n = 6/29)*	2 *(n = 2/12)*	4 *(n = 4/17)*	*2* *(n = 2/6)*	0 *(n = 0/0)*
*Chimeric SOFF*	23 *(n = 23/29)*	10 *(n = 10/12)*	13 *(n = 13/17)*	*4* *(n = 4/6)*	0 *(n = 0/0)*

*All flaps (DCIA = Deep Circumflex Iliac Artery Flap; FFF = Free Fibula Flap; SOFF = Scapula Osteocutaneous Free Flap) are listed. Also*,* the use of a skin paddle for DCIA and FFF or additionally the latissimus dorsi as chimeric flap for SOFF is indicated. Besides the number of the used flap*,* the rate of subtotal osseous union (number/total number of the flap type) and plate fracture (tnumber/total number of the flap type) is listed.*

**Table 2:** Group characteristics of CAD/CAM and conventional group

	Age (years ± SD)	Follow-up (years ± SD)	UICC CLASSIFICATION I-IV (*n/N*)	ASA classification I-IV (*n/N*)	Smoking (*n/N*)	Adjuvant therapy (*n/N*)
CAD/CAM	64.89 ± 10.41	1.04 ± 1.32	I (3/54) II (13/54) III (8/54) IV (30/54)	ASA I (0/56) ASA II (12/56) ASA III (42/56) ASA IV (2/56)	Yes (32/56) No (24/56)	Yes (11/56) No (45/56)
Conventional	58.66 ± 9.15	7.03 ± 5.63	I (9/52) II (6/52) III (12/52) IV (25/52)	ASA I (1/56) ASA II (23/56) ASA III (31/56) ASA IV (1/56)	Yes (40/56) No (16/56)	Yes (12/56) No (44/56)

*The age of all patients in years (± SD)*,* the follow up period in years (± SD)*,* UICC classification for all 106 OSCC patients the ASA classification (I-IV since V and VI were not given in any patient)*,* smoking (yes/no) and adjuvant therapy (yes/no) are listed. All as the proportion of all included cases.*

### Bone grafts

Of all 112 bone grafts (Table 1), the DCIA was used in 43 cases (DCIA: 43/112), followed by the FFF with 40 cases (FFF: 40/112) and the SOFF with 29 patients (SOFF: 29/112). It was found that the DCIA was the most frequently used graft in the CAD/CAM-group with 31 cases (DCIA CAD/CAM: 31/56), followed by the FFF with 13 cases (FFF CAD/CAM: 13/56), and the SOFF with 12 cases (SOFF CAD/CAM: 12/56). In contrast, within the conventional group, the FFF graft was found to be mostly used with 27 cases (FFF conventional: 27/56), followed by the SOFF with 17 cases (SOFF conventional: 17/56), and the DCIA with 12 cases (DCIA conventional: 12/56).

### Resection margin status

Overall, 104 (*n =* 104/112) surgeries could be evaluated regarding the resection margin status. For CAD/CAM carried out cases (*n* = 54/104), no R2 -margin status was found, but 3 R1-margin status (R1: 3/54) and 51 R0-margin status (R0: 51/54) could be found. For conventional surgeries (*n* = 50/104), no R2-margin status, 5 R1-margin status (R1: 5/50) and 45 R0-resection margin status (R0: 45/50) were documented (Table 3), with no significant differences between both groups (*p* = 0.509).

**Table 3:** Surgery related characteristics of CAD/CAM and conventional group

	Surgery time (hours ± SD)	Ischemic time (minutes ± SD)	Perioperative transfusion (*n/N*)	Resection Margin Status R0, R1, R2 (*n*/N)	ICU (days ± SD)
CAD/CAM	12.33 ± 2.59	87.06 ± 42.40	Yes (26/56) No (30/56)	R0 (51/54) R1 (3/54) R2 (0/54)	6.34 ± 4.18
Conventional	11.04 ± 2.59	154.38 ± 62.23	Yes (23/55) No (32/55)	R0 (45/50) R1 (5/50) R2 (0/50)	4.02 ± 3.54

*The surgery time is given in hours (± SD)*,* the ischemic time in minutes (± SD)*,* the need for red blood cell transfusion is given as yes/no and the proportion of all included cases*,* the resection margin status (R0; R1; R2) as total number and the proportion of all included cases*,* and the length of intensive care treatment in days (± SD).*

### Osseous union

Adequate postoperative imaging was available in 78 of all 112 patients whereby subtotal osseous union was observed in 14 patients (subtotal osseous union: 14/78) (Table 1). Reconstruction using SOFF were most frequently afflicted by subtotal osseous union (*n* = 6/14), followed by DCIA (*n* = 5/14) and FFF (*n* = 3/14). When comparing the surgical technique alone, the use of CAD/CAM-technique did not have a statistically significant effect on the incidence of subtotal osseous union (*p* = 0.474) (Table 4).

### Postoperative intensive care treatment

Further investigation on the postoperative length of stay on intensive care unit (ICU) revealed a higher number of days for CAD/CAM carried out surgery cases (6.34 ± 4.18 days) when compared to conventional cases (4.02 ± 3.54 days) (Table 3). No significant differences (*p* = 0.905) (Table 4) were found when comparing surgical technique alone. However, a reconstruction with chimeric SOFF graft in combination with CAD/CAM-technique showed significant effect on longer ICU stay (*p* = 0.039; 8.10 days) (Table 4) compared to the other grafts (4.69 days for FFF; 4.50 days for SOFF; 6.75 days for DCIA without and 6.47 days for DCIA with skin paddle). Furthermore, older patients showed significant association for longer ICU stay (*p* = 0.021).

**Table 4:** Statistical analyses and influence of reconstruction (conventional vs. CAD/CAM) and confounding factors

Predictors	Est.	SE	*p*-value	Bonferroni-Holm corrected *p*-value	Lower 2.5% CI	Upper 2.5% CI
Resection margin status						
Reconstruction	−0.038	0.058	0.509	1.000	−0.152	0.075
Age	−0.001	0.003	0.619	1.000	−0.006	0.004
Osseus union						
Reconstruction	0.259	0.361	0.474	1.000	−0.449	0.967
SOFF	0.015	0.234	0.950	1.000	−0.444	0.474
Chimeric SOFF	−0.114	0.213	0.592	1.000	−0.532	0.303
DCIA	0.033	0.141	0.816	1.000	−0.244	0.310
DCIA with skin paddle	−0.039	0.162	0.809	1.000	−0.356	0.278
Smoking	−0.132	0.119	0.266	1.000	−0.365	0.101
Adjuvant therapy	0.050	0.150	0.739	0.739	−0.244	0.344
ASA-Score	−0.039	0.120	0.745	1.000	−0.274	0.196
Age	0.003	0.006	0.543	1.000	−0.008	0.014
ICU stay						
Reconstruction	0.098	0.817	0.905	1.000	−1.504	1.699
SOFF	−0.153	0.559	0.785	1.000	−1.249	0.943
Chimeric SOFF	**0.937**	**0.455**	**0.039**	**0.195**	**0.045**	**1.828**
DCIA	0.261	0.535	0.626	1.000	−0.788	1.310
DCIA with skin paddle	0.332	0.397	0.402	1.000	−0.446	1.110
Smoking	−0.226	0.362	0.532	1.000	−0.937	0.484
ASA-Score	0.040	0.274	0.883	1.000	−0.498	0.578
Age	**0.024**	**0.010**	**0.021**	**0.147**	**0.004**	**0.044**
UICC	0.087	0.097	0.374	0.374	−0.104	0.277
Ischemic time						
Reconstruction	**−0.890**	**0.332**	**0.007**	**0.049**	**−1.541**	**−0.238**
SOFF	−0.176	0.719	0.807	1.000	−1.585	1.234
Chimeric SOFF	0.075	0.413	0.856	1.000	−0.735	0.885
DCIA	0.078	0.388	0.842	1.000	−0.683	0.838
DCIA with skin paddle	−0.301	0.353	0.394	1.000	−0.994	0.391
Age	−0.017	0.011	0.115	0.460	−0.038	0.004
RBC requirement						
Reconstruction	0.167	0.375	0.657	1.000	−0.569	0.903
SOFF	**0.498**	**0.205**	**0.015**	**0.090**	**0.097**	**0.899**
Chimeric SOFF	−0.244	0.201	0.225	0.708	−0.638	0.15
DCIA	**0.395**	**0.189**	**0.036**	**0.216**	**0.026**	**0.765**
DCIA with skin paddle	0.2	0.193	0.298	1.000	−0.177	0.578
Smoking	−0.014	0.152	0.927	1.000	−0.311	0.283
ASA-Score	−0.094	0.123	0.444	1.000	−0.336	0.147
Age	**−0.01**	**0.005**	**0.035**	**0.210**	**−0.02**	**−0.001**
Surgical time						
Reconstruction	−0.599	0.716	0.403	1.000	−2.002	0.805
SOFF	−0.147	0.450	0.743	1.000	−1.028	0.734
Chimeric SOFF	**1.293**	**0.403**	**0.001**	**0.006**	**0.504**	**2.083**
DCIA	−0.132	0.359	0.714	1.000	−0.836	0.572
DCIA with skin paddle	0.231	0.278	0.407	1.000	−0.315	0.776
ASA-Score	0.261	0.245	0.286	1.000	−0.219	0.741
Age	**0.017**	**0.009**	**0.050**	**0.250**	**0.000**	**0.034**
UICC	−0.163	0.099	0.099	0.198	−0.357	0.031
Plate fracture						
Reconstruction	0.065	0.15	0.667	1.000	−0.230	0.359
SOFF	−0.196	0.165	0.235	1.000	−0.519	0.128
Chimeric SOFF	−0.134	0.099	0.177	0.708	−0.329	0.061
DCIA	−0.098	0.145	0.500	1.000	−0.382	0.186
DCIA with skin paddle	−0.181	0.121	0.135	0.810	−0.419	0.056
Smoking	−0.058	0.098	0.553	1.000	−0.251	0.134
Adjuvant therapy	0.147	0.113	0.194	0.388	−0.075	0.368
Age	−0.004	0.003	0.201	0.603	−0.010	0.002

*Confounding factors regarding osseus union*,* ICU stay*,* RBC requirements*,* surgical time*,* plate fracture*,* resection margin status and ischemic time. Statistically significant values are marked in bold. Different flap types are listed as DCIA = Deep Circumflex Iliac Artery Flap; FFF = Free Fibula Flap; SOFF = Scapula Osteocutaneous Free Flap.*

### Ischemic time

A total of 60 patients (Available for ischemic time analysis: 60/112) allowed the clear evaluation of the documented ischemic time, including 24 patients of the conventional group (Ischemic time conventional group: 24/60) and 36 of the CAD/CAM group (Ischemic time CAD/CAM group: 36/60) (Table 3). Here, the ischemic time was found to be significantly lower (*p* = 0.007) in the CAD/CAM group (87.06 min) when compared to the conventional group (154.38 min) (Table 4).

### Perioperative red blood cell transfusion

Most cases (*n* = 111/112) allowed an investigation on perioperative administration of red blood cell transfusion (RBT). Of all 56 CAD/CAM cases, transfusion was required in 26 (RBT: 26/56) patients, whereas transfusion was only required in 23 of 55 conventional cases (RBT: 23/55) (Table 3). Nevertheless, no significant differences comparing both groups alone could be found (*p* = 0.657). Statistical analyses revealed significant effect (*p* = 0.015 and 0.036 respectively) (Table 4) on increased requirement of RBT when CAD/CAM-technique is combined with a SOFF or a DCIA graft.

### Surgical time

The surgical time in hours was found to be comparable within both groups with 12.33 ± 2.59 h for the CAD/CAM group and 11.04 ± 2.59 h for the conventional group (Table 3). No significant differences (*p* = 0.403) comparing both groups alone could be found. Reconstruction with chimeric SOFF graft in combination with CAD/CAM showed a significant effect on longer surgical time (*p* = 0.001; 15.10 h) compared to other grafts (FFF = 11.42 h; non-chimeric SOFF = 12.00 h; DCIA = 11.77 h) (Table 4). Furthermore, older patients showed significant association for longer surgical time (*p* = 0.050).

### Plate fracture

In a total of 9 cases plate fracture (plate fracture: 9/112) was noted (Table 1), whereby 3 plate fractures were observed in the CAD/CAM group (plate fracture: 3/56) compared to the conventional group (plate fracture: 6/56). However, no significant differences comparing both groups alone could be found (*p* = 0.667).

## Discussion

Within the last decade, CAD/CAM assisted osseous reconstruction of the head and neck region has been implemented in the routine workflow of most departments of oral and maxillofacial surgery [27, 28]. The CAD/CAM assisted workflow already proofed to result in higher accuracy [36–38], achieving high esthetic and functional results. Nevertheless, most studies reporting advantages connected to CAD/CAM osseous reconstruction in the head and neck, only include a relatively low number of patients on average [29]. Therefore, despite the high number of existing publications, this study aims to compare a high number of patients who received osseous reconstruction of the head and neck region, either through a conventional, or a CAD/CAM bone graft. The study population included not only reconstructions using FFF [39, 40], but also DCIA, and SOFF. Despite its standing as the “workhorse flap”, there are several reasons for using a different bone graft than the FFF. Dowthwaite et al., consider free flaps of the subscapular system as excellent options for elderly patients, with peripheral vascular disease, and mandible defects associated with complex soft-tissue requirements [41]. The SOFF might also offer advantages in osseous head and neck reconstructions by facilitating non-osteotomized and chimeric reconstructions without compromising surgical outcomes or quality of life [42]. This was also observed in our study population, as in 23 of 29 SOFF cases, the latissimus dorsi muscle was included in the transplant as a chimeric flap due to the extent of the defect after tumor resection. In this study, patients receiving a CAD/CAM chimeric SOFF showed a significant effect on longer surgical time and a significant longer ICU stay^1^. A possible reason for this might be, that patients receiving CAD/CAM-surgery were found to be significantly older and that the chimeric SOFF was mostly used to reconstruct defects associated with complex soft-tissue requirements. This is also reflected in the higher number of ASA III and IV classification, as well as the higher number of UICC IV classification cases in the CAD/CAM group (Table 2). Another reason for the longer surgery time might be due to intraoperative repositioning of the patients when compared to patients receiving a DCIA or FFF.

In accordance with national guidelines for the resection of OSCC [43, 44], resection margins must be tumor-free and maintain a safe distance from the tumor. In CAD/CAM reconstruction, high-resolution CT scans are typically evaluated, and virtual surgical planning is conducted before the operation [17]. During surgery, guided techniques may limit the surgeon’s ability to deviate from the planned resection [45]. If a more extensive osseous resection becomes necessary, it could render the CAD/CAM reconstruction unfeasible, even if the soft tissue resection remains unaffected [46]. Previous studies on involved resection margins (microscopic tumor at resection margin) vary widely, whereas a large cohort study had an incidence of 14.6% regarding microscopic tumor at the resection margin [47]. In our study, a R0 margin status could be achieved in 94.4% of the CAD/CAM cases, which was higher when compared to the conventional group (90.0%). These findings align with the study of Goetze et al. who already demonstrated that the resection of OSCC combined with primary CAD/CAM-based reconstruction enables safe tumor removal [46].

Recent studies hint towards a clear trend for shorter overall survival and tumor-free survival for patients who received RBT during primary surgery of OSCC [48–50]. A non-head and neck related study dealing with the influence of the CAD/CAM technique on red blood cell transfusion revealed e.g. a significantly lower volume of blood loss, lower blood transfusion volume, lower fresh frozen plasma volume in infants with complex craniostenosis following fronto-orbital advancement when compared to the conventional method [51]. In contrast, a comparable study about CAD/CAM vs. conventional carried out surgery of trigonocephaly in children showed a higher amount of blood transfusion for the CAD/CAM group (80.0% vs. 70.0%) [52]. To the best of the authors knowledge, no study evaluated the need for RBT in oncological head and neck surgeries with special emphasize on the use of CAD/CAM bone flaps. Here, RBT was required in 46.43% patients of the CAD/CAM group, and only 41.82% of the conventional cases. The higher number of RBT in CAD/CAM patients may be due to the older study population (conventional: 58.66 ± 9.15 years vs. CAD/CAM: 64.98 ± 10.41 years) since Dejam et al. showed that older and frail patients benefit from RBT [53]. Moreover, the higher number ASA classification II and IV, and the higher number of UICC classification IV cases in the CAD/CAM group needs to be taken into account. It should also be considered that the use of resection and drilling guides may require longer skin incisions to allow more space for the bulky guides to be inserted into the donor site/resection site.

When comparing conventional and CAD/CAM techniques in osseous reconstruction with FFF, Rustemeyer et al. showed that ischemia was significantly lower in the CAD/CAM group [54], which is in line with our findings. Nonetheless, mean surgical time was found to be almost one hour longer for the CAD/CAM group (12.33 ± 2.59 h vs. 11.04 ± 2.59 h). In addition to the already mentioned older study population of the CAD/CAM group, the higher number of UICC classification IV patients in the CAD/CAM group and the use of a chimeric SOFF for complex reconstructions may have required more extensive and time-consuming reconstructions as in the conventional group. The same applies for the longer ICU stay of CAD/CAM patients and especially patients with chimeric SOFF reconstruction in our group, which contrasts with other studies [55] but might be explained by the higher age of the CAD/CAM patients. As CAD/CAM allows for advanced surgical planning, more complex reconstructions are being chosen compared to conventionally operated patients. This may also explain the longer operating time and ICU stay, as a more extensive surgical approach is required.

Our study also determined the rate of osseous union and compared it between both groups. Here, the subtotal osseous union rate was higher in the conventional group, which was not found to be significant. In contrast, Knitschke et al. demonstrated a lower rate of incomplete osseous union (IOU) for the non-PSI group (vs. PSI group) in the long-term. After 36 months, only 4.2% of non-PSI cases showed IOU, compared to 18.2% in the PSI group [56]. Despite the shorter follow up of CAD/CAM patients in our group, our findings indicate a reduced number of subtotal osseous union (10.71%) in the CAD/CAM group. When compared to other studies, our data on subtotal osseous union seems to be relatively low. One reason for the lower rate of subtotal osseous union in CAD/CAM patients, could be the higher stability of PSIs, which might be responsible for the better healing compared to the conventional cases in our study group. Nevertheless, the discussion, if a more flexible construct can lead to an accelerated bone healing remains controversial [56, 57]. Bartnikowski et al. have shown in an animal model that a stiffer construct is advantageous overall, especially in the second phase of bone healing, because a stiffer construct leads to more predictable bone healing results. Additionally, higher stability of PSIs can reduce the risk of plate fracture, which is in line with our study, since microcracks in manually bend plates may increase the risk of plate fracture due to plate-surface damage from bending tools and to alterations in internal metal structure [58]. Nevertheless, regarding plate-related complications, it should be emphasized that the follow-up period for CAD/CAM patients was significantly shorter, representing a clear limitation of this study. For instance, Yang et al. reported a mean time to plate removal of 17.4 months following CAD/CAM reconstruction, with plate exposure and screw loosening being the primary indications for removal [59]. In comparison, the mean follow-up duration in our CAD/CAM cohort was only 1.04 ± 1.32 years, underscoring the need for further prospective investigations to elucidate potential differences between CAD/CAM and conventional osseous reconstruction.

### Limitations

Although this study included a large cohort of patients who underwent osseous reconstruction (both CAD/CAM and conventional) of the head and neck region, the sample size remains insufficient to generalize the results. The ASA-score was considered in order to generally include the health status of the patients in the study, nevertheless, several specific health-related factors (e.g., cardiovascular diseases, diabetes mellitus) and numerous periprocedural factors (e.g., the use of microscopes, coupler devices, suture techniques, and surgical approaches to soft-tissue management) were not accounted for in this study. Moreover, an imbalance between the shorter follow-up time of the CAD/CAM group compared to the conventional group exists, since CAD/CAM technology was introduced at a later stage, and used in older and more compromised patients. In addition, these patients had a higher UICC stage, which increases the likelihood of death and thus also influences the follow-up duration. That may impact the outcomes, especially late complications like osteoradionecrosis and osseous non-union [60–62]. In contrast to that, complications like (fatigue) plate fractures tend to occur within the first 6 months [23]. Since the follow-up time of the CAD/CAM-patients exceeds this period, it is likely that the unevenly dispensed sample might not have caused any bias here. The potential influence of these factors may confound the true impact of CAD/CAM surgery, underscoring the need for cautious interpretation of the presented findings.

## Data Availability

Data available on request due to privacy and ethical restrictions.

## Footnotes

1 In our evaluation we focused on non-corrected p-values instead of Bonferroni-Holm-corrected p-values, as our focus lies on the explanatory nature. The probability of every single predictor should be evaluated by itself. Nevertheless Bonferroni-Holm-corrected p-values are reported in Table 4.
